# Identification of miRNomes reveals ssc-miR-30d-R_1 as a potential therapeutic target for PRRS viral infection

**DOI:** 10.1038/srep24854

**Published:** 2016-04-27

**Authors:** Chengmin Wang, Yanyu Zhang, Jing Luo, Hua Ding, Shelan Liu, Said Amer, Li Xie, Wenting Lyv, Wen Su, Meng Li, Qinmiao Sun, Jiayin Dai, Hongxuan He

**Affiliations:** 1 Key Lab of Animal Ecology and Conservation Biology, Institute of Zoology, Chinese Academy of Sciences, Beijing, 100101, P.R China; 2 Beijing Institute of Transfusion Medicine, Academy of Military Medicine Sciences, Beijing, 100850, P.R China; 3 Department of Infectious Disease, Hangzhou Center for Disease Control and Prevention, Hangzhou, Zhejiang Province, 310021, P.R China; 4 Department of Infectious Diseases, Zhejiang Center for Disease Control and Prevention, Hangzhou, Zhejiang Province, 310051, P.R China; 5 Department of Zoology, Faculty of Science, Kafr El sheikh University, Kafr El sheikh 33516, Egypt

## Abstract

Porcine reproductive and respiratory syndrome virus (PRRSV) is known to cause reproductive disorders, such as abortion, in pregnant sows as well as immunosuppressive respiratory complications, leading to severe respiratory tract infections in young pigs. In this study, an in-depth analysis of the miRNomes in mock- and virus-infected pig lungs was carried out. We found that highly expressed ssc-miR-30d-R_1 was decreased in infected lungs, and reduced levels were significantly correlated with infection by PRRSV. Moreover, ssc-miR-30d-R_1 was shown to target Toll-like receptor 4 (TLR4) and to suppress the production of immune cytokines through inhibition of the TLR4/MyD88/NF-κB pathway. ssc-miR-30d-R_1 significantly reduced viral infections and pathological changes in pig lungs *in vivo*. Our current study reveals the miRNomes of PRRSV-infected pig lungs and indicates that ssc-miR-30d-R_1 is potential therapeutic agent for controlling PRRSV infection.

The discovery of host microRNA (miRNA) targets in the genomes of many vertebrate viruses indicates that the corresponding miRNAs are a part of the host’s innate antiviral defenses. miRNAs are post-transcriptional regulators that bind to complementary sequences on target messenger RNA transcripts (mRNAs), resulting in translational repression and gene silencing^1^. miRNAs are considered to be a major group of functional non-coding RNAs (ncRNAs) that bind to mRNAs, resulting in target-specific post-transcriptional repression^2^. Growing evidence indicates that miRNAs can directly modulate viral replication and modify host cell responses to viral infection in a proviral or antiviral manner^3^,^4^.

Porcine reproductive and respiratory syndrome virus (PRRSV), which belongs to the *Arteriviridae* family, causes one of the most economically devastating diseases affecting the swine industry worldwide^5^. It induces reproductive disorders, such as abortion, in pregnant sows, as well as severe respiratory tract disease in young pigs^6^,^7^. PRRSV replicates mainly in porcine alveolar macrophages (PAMs) and dendritic cells (DCs) and leads to persistent infection, interstitial pneumonia and immunosuppression. Growing evidence has indicated that miRNAs play important roles in regulating viral infections^3^,^4^. However, there are few studies that have focused on the interaction between PRRSV and miRNAs. Wang *et al.*^8^ reported that miR-125b reduced PRRSV replication by negatively regulating the NF-κB pathway, and miR-24-3p promoted PRRSV replication through suppression of heme oxygenase-1 expression^9^. On the other hand, miR-181 inhibited PRRSV replication by targeting both viral genomic RNA and receptor CD163^10^,^11^.

The present study aimed to analyze the porcine miRNomes expressed during mock infection and the early stages of infection using deep sequencing technology. Furthermore, we identified the miRNomes pre- and post-infection with PRRSV strain LS-4 and concluded that ssc-miR-30d-R_1 targeted Toll-like receptor 4 (TLR4) of MARC-145 cells to suppress the production of immune cytokines by inhibiting the TLR4/MyD88/NF-κB pathway both *in vitro* and *in vivo*. This study described the miRNomes of PRRSV in infected pig lungs and may contribute to understanding the miRNA involvement in the porcine immunosuppressive response during PRRSV infection.

## Results

### The miRNomes in control and PRRSV-infected swine lung tissues

MPSS (massively parallel signature sequencing) was used to analyze the miRNomes of three mock-infected and three PRRSV-infected lung tissue samples. The abundance value of each known miRNA was normalized using transcripts per million (TPM) in each small RNA library. The results showed that in normal lung tissues, 76.5% of miRNAs were poorly expressed (<10 TPM), 19.1% of miRNAs were modestly expressed (10–1000 TPM), and 3.59% of miRNAs showed moderately high expression (1000–10000 TPM), whereas only 0.79% of miRNAs were highly expressed (>10000 TPM). In infected lung tissues, 74.4% of miRNAs were poorly expressed (<10 TPM), 20.67% of miRNAs were modestly expressed (10–1000 TPM), and 4.13% of miRNAs showed moderately high expression (1000–10000 TPM), whereas only 0.78% of miRNAs were highly expressed (>10000 TPM). In total, seventy-two expressed miRNAs had TPM <10000 and ≥1.0-fold change, and several selected miRNAs (TPM >100 and fold >1.5) were used for further analysis (Table 1).

### Downregulated expression of ssc-miR-30d_R-1 in PRRSV-infected PAM and MARC-145 cells

**Figure 1:**
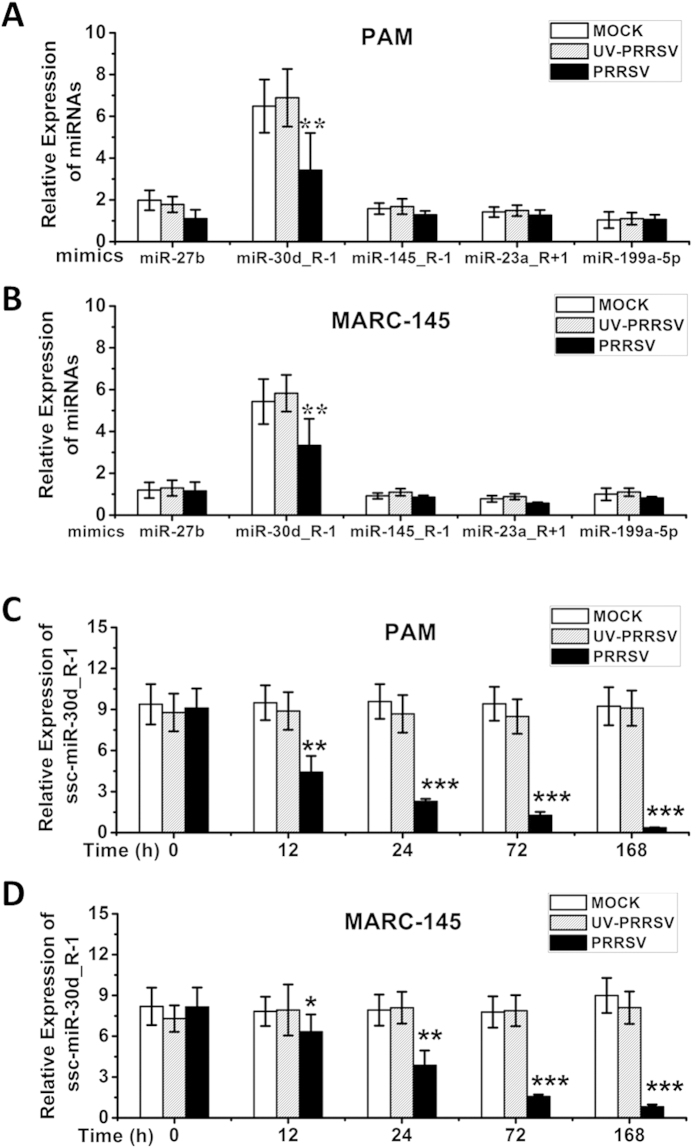
PRRSV infection inhibits ssc-miR-30d_R-1 expression in primary cultured porcine alveolar macrophages (PAMs) and MARC-145 cells. Cells were infected with PRRSV strain LS-4 (MOI of 1.0), and real-time qRT-PCR analysis of ssc-miR-30d_R-1, ssc-miR-27b, ssc-miR-145_R-1, ssc-miR-23a_R + 1, and ssc-miR-199a-5p expression was carried out in mock-, UV-PRRSV- and PRRSV-infected primary cultured PAMs (**A,C**) and MAC-145 cells (**B,D**). Data are expressed as the mean ± standard deviation of three independent experiments. P values were calculated using a Student’s t test. **P < 0.01; ***P < 0.001 (relative to the mock control).

Few miRNAs were abundantly expressed in the miRNomes, and they appeared to be important in the interaction between the lungs and PRRSV infection. Only miRNAs with TPM >1000 and more than a 1.5-fold alteration were considered likely to be important in PRRSV pathogenesis in this study (Table 1). Together, the miRNomes of mock- and PRRSV-infected piglet lungs enabled identification of downregulated miRNAs in PRRSV-infected MARC-145 and PAM cells. As shown in Fig. 1, the abundant miRNAs in PRRSV-infected cells were further confirmed by qRT-PCR. ssc-miR-30d_R-1 expression was the most consistently and markedly decreased in both PAM cells (Fig. 1A) and MARC-145 cells (Fig. 1B) compared to matched mock controls and UV-PRRSV. However, the changes in other miRNAs—ssc-miR-27b, ssc-miR-145_R-1, ssc-miR-23a_R + 1 and ssc-miR-199a-5p—in PRRSV infection were found to be relatively small in infected PAM (Fig. 1A) and MARC-145 cells (Fig. 1B).

To determine whether PRRSV infection affects the expression of ssc-miR-30d_R-1, ssc-miR-30d_R-1 levels in the PAM and MARC-145 cells were quantified by qPCR at different time points after PRRSV infection. As shown in Fig. 1C,D, ssc-miR-30d_R-1 showed pronounced and progressive downregulation in both PAM and MARC-145 cells infected with PRRSV as early as 12 h post infection compared to the mock control. In addition, PRRSV inactivated by UV irradiation did not alter ssc-miR-30d_R-1 expression, indicating the importance of ssc-miR-30d_R-1 in the interaction between cells and PRRSV infection (data not shown).

### ssc-miR-30d_R-1 inhibits PRRSV replication

**Figure 2:**
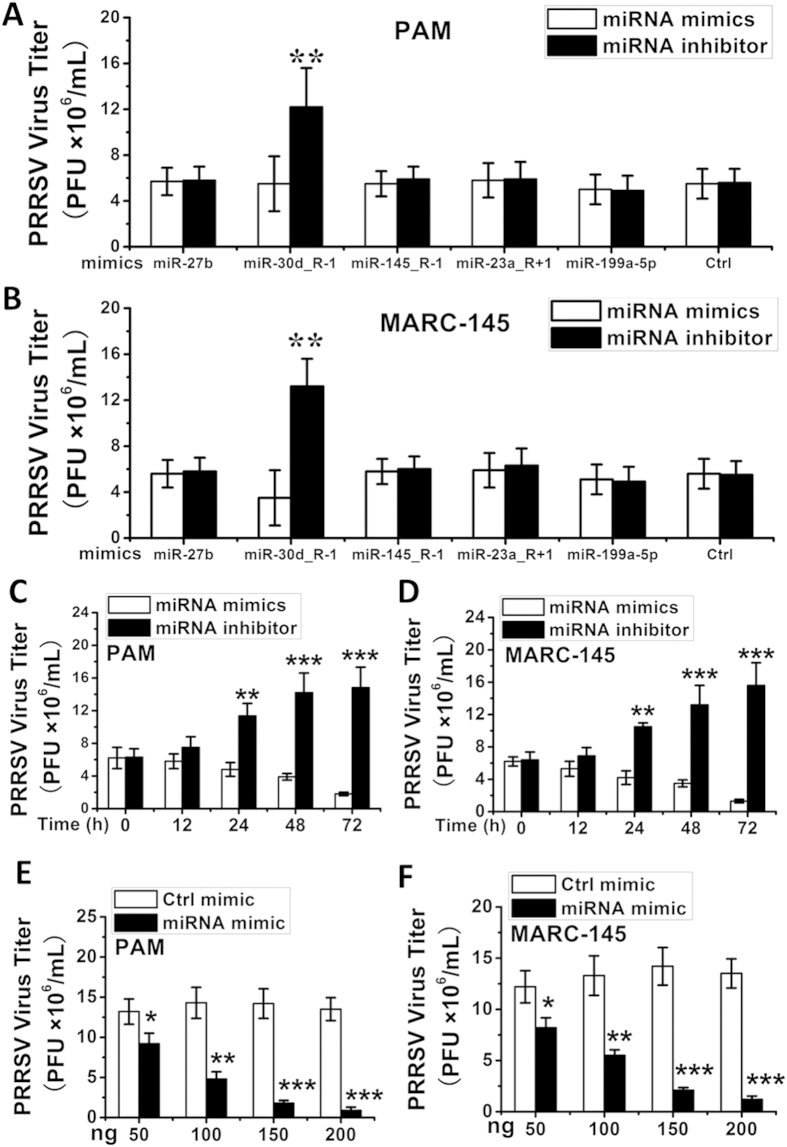
ssc-miR-30d_R-1 reduces PRRSV replication in MARC-145 cells and PAMs. Expression of ssc-miR-30d_R-1, ssc-miR-27b, ssc-miR-145_R-1, ssc-miR-23a_R + 1, and ssc-miR-199a-5p mimics and inhibitors in PAM cells (**A**) and MARC-145 cells (**B**). Expression of the ssc-miR-30d_R-1 mimic reduced PRRSV replication in a time-dependent manner in PAM cells (**C**) and MARC-145 cells (**D**). Expression of the ssc-miR-30d_R-1 mimic reduced PRRSV replication in a dose-dependent manner in PAM cells (**E**) and MARC-145 cells (**F**). MARC-145 and PAM cells were transfected with the ssc-miR-30d_R-1 mimic or a control mimic at the indicated dose (50,100,150 and 200 ng), followed by PRRSV infection (MOI = 0.1). Data are expressed as the mean ± standard deviation of three independent experiments. P values were calculated using a Student’s t test. *P < 0.05; **P < 0.01; ***P < 0.001.

A panel of 5 miRNA mimics or inhibitors of various miRNAs, including ssc-miR-30d_R-1, ssc-miR-27b, ssc-miR-145_R-1, ssc-miR-23a_R + 1, and ssc-miR-199a-5p, were synthetized to ascertain their impact on PRRSV replication. MARC-145 and PAM cells were transfected with the mimic or inhibitor of each miRNA (100 ng), followed by infection with PRRSV (LS-4 strain) at an MOI of 0.1. Cells were collected at 48 h post infection to determine the level of viral propagation. Among the miRNAs tested, the ssc-miR-30d_R-1 mimic significantly reduced progeny PRRSV production as determined by viral titer (PFU, ×10^6^ mL) (Fig. 2A). Conversely, transfection of the ssc-miR-30d_R-1 inhibitor counteracted these effects (Fig. 2B), indicating that ssc-miR-30d_R-1 has antiviral activity against PRRSV replication. All the other microRNA mimics/inhibitors tested in this study had undetectable effects on progeny virus yield in MARC-145 and PAM cells (Fig. 2A,B). However, we could not exclude the possibility that the undetectable effects of the other miRNA mimics or inhibitors may be due to the high/low expression of endogenous miRNAs.

To determine whether ssc-miR-30d_R-1 can inhibit PRRSV replication, MARC-145 and PAM cells were transfected with the ssc-miR-30d_R-1 mimic or inhibitor (100 ng), followed by infection with PRRSV (LS-4 strain) at an MOI of 0.1. Cells were collected at 0, 12, 24, 48 and 72 h post infection to determine viral propagation. As shown in Fig. 2C,D, viral titer in mimic-transfected cells was markedly and progressively decreased in MARC-145 and PAM cells infected with PRRSV as early as 12 h up to 72 h post infection compared to matched inhibitor-transfected cells.

To determine the dose effect of ssc-miR-30d_R-1 on PRRSV replication in PAM and MARC145 cells, the cells were transfected with increasing concentrations of ssc-miR-30d_R-1 mimic and control mimic (50, 100, 150 and 200 ng), followed by PRRSV infection 48 h after transfection. Viral plaque assays demonstrated that ectopic expression of the ssc-miR-30d_R-1 mimic reduced PRRSV replication in a dose-dependent manner in PAM cells compared to the control (Fig. 2E). Consistent with these findings, the ssc-miR-30d_R-1 mimic dose-dependently reduced the accumulation of PRRSV in MARC-145 cells (Fig. 2F).

To exclude the possibility that the inhibitor effects of ssc-miR-30d_R-1 on PRRSV replication resulted from cellular toxicity, MARC-145 and PAM cells were transfected with the ssc-miR-30d_R-1 mimic or control mimic at different doses (50, 100, 150 and 200 ng). No appreciable effect of ssc-miR-30d_R-1 (up to 200 ng) on cellular viability and morphology was observed (data not shown). Collectively, these data unequivocally confirm that ssc-miR-30d_R-1 inhibited PRRSV replication.

### ssc-miR-30d_R-1 does not directly target the PRRSV genome

To verify the specific target of ssc-miR-30d_R-1 in the PRRSV genome, 21 cDNA fragments representing the 5′-UTR, 3′-UTR and various coding regions of the PRRSV genome were amplified and cloned into the reporter vector pMIR-REPORT (Ambion) downstream of the firefly luciferase gene (Fig. S1). The results indicated that the relative luciferase activity of different vectors containing various PRRSV cDNA segments at 24 h post transfection did not differ significantly in cells transfected with the ssc-miR-30d_R-1 mimic compared to those transfected with the mimic controls (Fig. S1). Thus, ssc-miR-30d_R-1 does not appear to directly target the PRRSV genome.

### ssc-miR-30d_R-1 downregulates expression of pro-inflammatory cytokines *in vitro*

**Figure 3:**
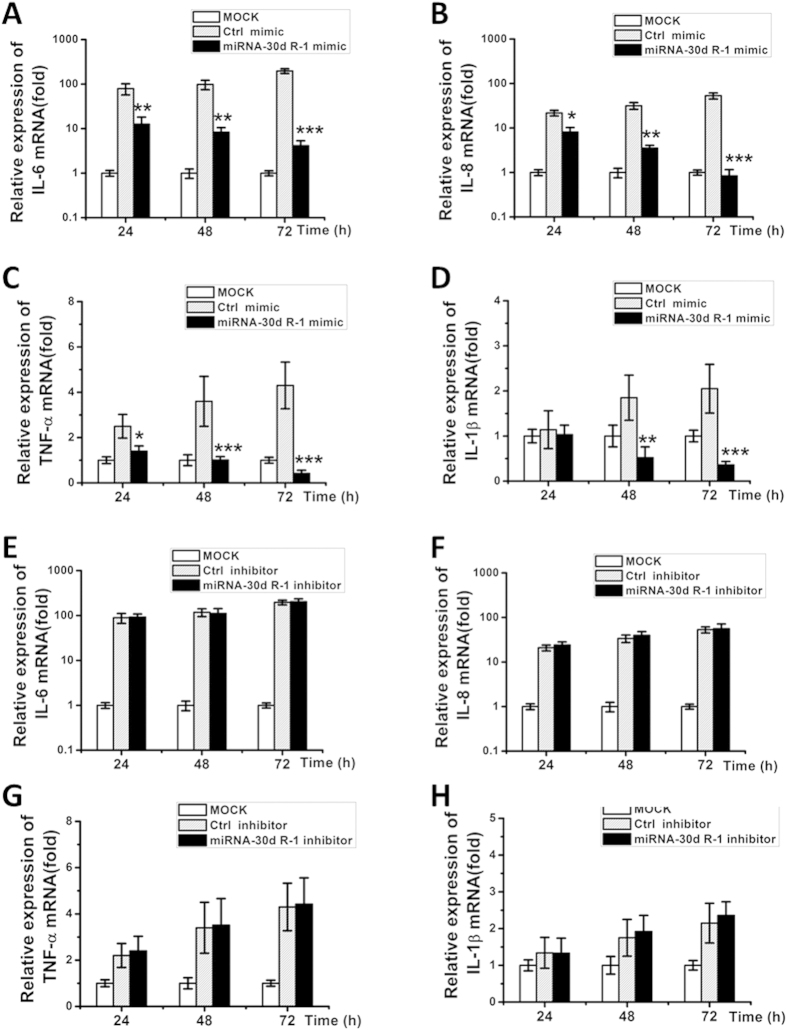
The suppressive effect of ssc-miR-30d_R-1 on pro-inflammatory cytokines. (**A–D**) indicate overexpression of the ssc-miR-30d_R-1 mimic and control mimic in PAMs. The ssc-miR-30d_R-1 mimic reduced mRNA levels of pro-inflammatory cytokines (IL-8, IL-6, TNF-α and IL-1β) in a time-dependent manner in PAM cells. (**E–H**) indicate ssc-miR-30d_R-1 inhibitor-enhanced mRNA levels of pro-inflammatory cytokines (IL-8, IL-6, TNF-α and IL-1β) in a time-dependent manner in PAM cells (**F**). PAM cells were transfected with mimics or inhibitors, followed by PRRSV infection (MOI = 0.1). Data are expressed as the mean ± standard deviation of three independent experiments. P values were calculated using a Student’s t test. *P < 0.05; **P < 0.01; ***P < 0.001. Similar results in MARC-145 cells are shown in Fig. S2.

To verify the regulation of pro-inflammatory cytokines by ssc-miR-30d_R-1 in PRRSV-infected PAM cells, mRNA levels of IL-1β, IL-6, IL-8 and TNF-α in mock-, miRNA mimic- or inhibitor-transfected and control mimic- or inhibitor-transfected cells were evaluated. At 48 and 72 h post infection, mRNA expression of IL-1β, IL-6, IL-8, and TNF-α in mimic-transfected cells was substantially decreased compared to the control mimic (Fig. 3A–D). Correspondingly, mRNA levels of the listed cytokines did not differ significantly between miRNA inhibitor- and control inhibitor-transfected cells (Fig. 3E–H). Similar results were also observed in MARC-145 cells (Fig. S2). Taken together, the ssc-miR-30d_R-1 mimic can regulate the expression of pro-inflammatory cytokines.

### ssc-miR-30d_R-1 directly targets the 3′-UTR of TLR4

**Figure 4:**
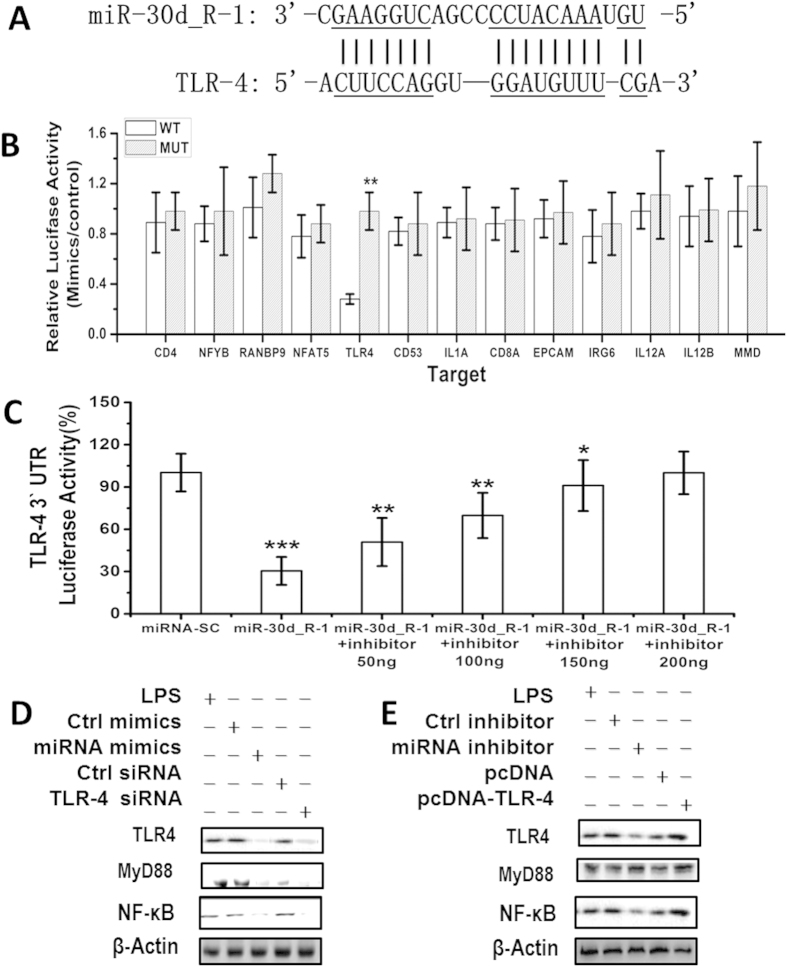
Direct targeting of TLR4 mRNA by ssc-miR-30d_R-1. The predicted conserved ssc-miR-30d_R-1-binding site (underlined) in the 3′-UTR of pig TLR4 mRNA (**A**). Luciferase activity in MARC-145 cell lysates transfected with constructs encoding wild-type (WT) or mutated (Mut) target gene 3′-UTRs plus mimics (**B**) or inhibitors (**C**) of ssc-miR-30d_R-1 (or the appropriate control). Validation of ssc-miR-30d_R-1 targeting of the TLR4 3′-UTR by treatment with different doses of inhibitor at 48 h after transfection (**D**). *P < 0.05 and **P < 0.01 (Student’s t test). Data are from three independent experiments (mean ± SD). (**E**) MARC-145 cells transfected with ssc-miR-30d_R-1 (miRNA) mimic or inhibitor, control siRNA, pc-DNA-TLR4 or TLR4 siRNA plasmids were stimulated by LPS. Expression of β-actin (internal control), TLR4, MyD88, and NF-κB were detected by Western blot. Data shown are representative of three independent experiments.

Thirteen target genes for ssc-miR-30d_R-1 that have a role in PRRSV pathogenesis were predicted using the *Sus scrofa* Unigene database (NCBI) and the miRanda algorithm (version 3.3; http://www.microrna.org) (Table S3). The 3′-UTR of the TLR4 mRNA contains the ssc-miR-30d_R-1-binding site (Fig. 4A). We used the reporter gene system to verify the database predictions. The results indicated that the ssc-miR-30d_R-1 mimic (synthetic miRNAs that mimic the function of endogenous ssc-miR-30d_R-1) significantly inhibited the luciferase activity of the TLR4 3′-UTR reporter but did not affect the luciferase activity of other possible target gene reporters with mutated ssc-miR-30d_R-1-binding sites (Fig. 4B). Although ssc-miR-30d_R-1 was able to significantly suppress luciferase activity after the addition of 50 or 100 ng of ssc-miR-30d_R-1 inhibitor, adding 150 or 200 ng of ssc-miR-30d_R-1 inhibitor did not reverse this suppression trend (Fig. 4C), indicating that the endogenous TLR4 was targeted and regulated by ssc-miR-30d_R-1.

### ssc-miR-30d_R-1 inhibits the TLR4/MyD88-dependent signaling pathway

To identify the role of TLR4 and ssc-miR-30d_R-1 in PRRSV pathogenesis, the TLR4 gene was subjected to enrichment analysis of cell signaling pathways using the Kyoto Encyclopedia of Genes and Genomes (KEGG) pathway database (http://www.genome.jp/kegg/). Analysis results indicate that the nuclear factor κB (NF-κB) signaling pathway was the most enriched of the predicted targets of ssc-miR-30d_R-1. Activation of NF-κB is known to play critical roles in PRRSV replication^12^.

**Figure 5:**
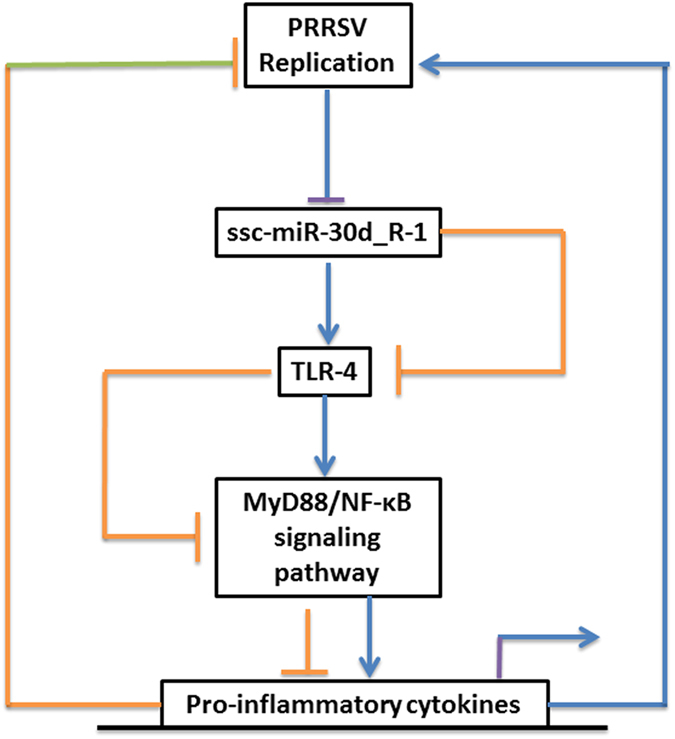
The possible mechanism of the suppressive effect of ssc-miR-30d_R-1 on PRRSV replication. PRRSV infection downregulates the expression of ssc-miR-30d_R-1 (targets TLR4), which activates the TLR4/MyD88/NF-κB signaling pathway, resulting in promotion of PRRSV replication. Conversely, overexpression of the ssc-miR-30d_R-1 mimic inhibits TLR4, which inhibits the TLR4/MyD88/NF-κB signaling pathway, resulting in reduced PRRSV replication.

The hypothesis that the TLR4/MyD88-dependent signaling pathway was involved in the antiviral effect of ssc-miR-30d_R-1 by targeting TLR4 was verified. The results showed ssc-miR-30d_R-1 and TLR4 siRNA downregulated the expression of MyD88 (myeloid differentiation primary response gene 88) and inhibited the activation of NF-κB in MARC-145 cells (Fig. 4D). Moreover, inhibition of ssc-miR-30d_R-1 or pcDNA TLR4 also enhanced the activation of the NF-κB pathway in MARC-145 cells (Fig. 4E), indicating that ssc-miR-30d_R-1 can inhibit the TLR4/MyD88-dependent signaling pathway to suppress PRRSV pathogenesis (Fig. 5).

### Inhibitory role of ssc-miR-30d_R-1 on viral replication in PRRSV-inoculated SPF piglets

**Figure 6:**
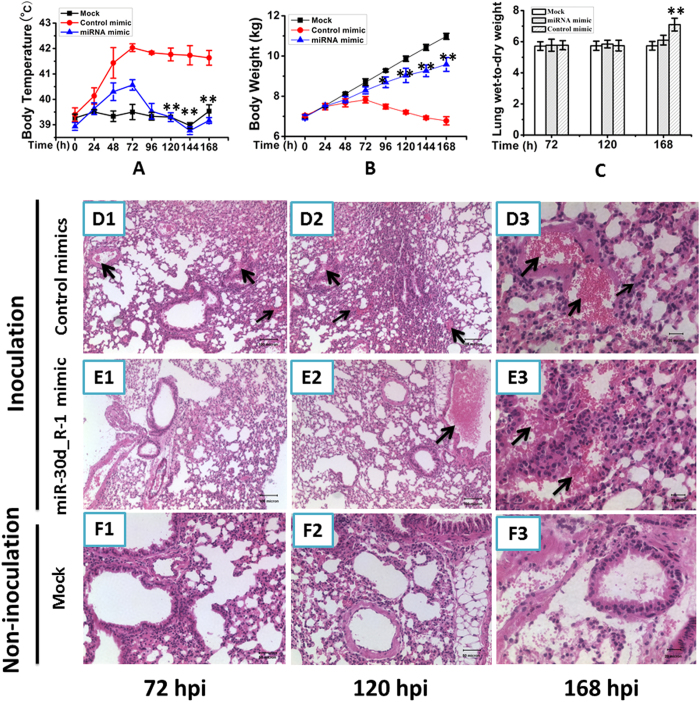
ssc-miR-30d_R-1 suppressed the pathogenic effect of PRRSV in SPF piglets. The piglets were treated with PBS, control mimic and synthetic ssc-miR-30d_R-1 mimic before being inoculated with PRRSV. The body temperature (**A**) and body weight (**B**) were measured at every time point. At 72, 120 or 168 h post inoculation, the piglets were sacrificed, and lung tissue was obtained to calculate the lung wet:dry weight ratio (**C**) and measure the pathological changes in the lungs (**D–F**, inflammatory cells infiltration: solid and thick arrows and hyperemia: solid and thin arrows). *P < 0.05; **P < 0.01. All values are expressed as the mean ± SD. P values were determined using a Student’s t test by comparing to the virus only group.

The antiviral effect of ssc-miR-30d_R-1 was tested in PRRSV-infected SPF piglets. Four-week-old female SPF piglets were intravenously administered the synthetic ssc-miR-30d_R-1 mimic (0.1 nmol per day) one day before being inoculated with 10^5^ TCID_50_ PRRSV LS-4 strain. The piglets in the control mimic-infected group showed severe clinical symptoms. At 24 h post infection, all the piglets started to develop elevated body temperatures (>40 °C) with a peak of 41.9 °C at 72 h post infection, but a peak of 40.5 °C at 72 h post infection was observed in piglets from the miRNA mimic-infected group (Fig. 6A). The body weight gain in piglets from the control mimic-infected group was reduced compared with the mock- and miRNA mimic-infected groups (Fig. 6B). The lung wet:dry weight ratio of the control mimic-infected piglets was higher than that of the miRNA-infected pigs, with a significant difference at 168 h post infection (p < 0.01) (Fig. 6C). The histopathological changes in lungs of the infected piglets were observed at 72, 120 and 168 h following inoculation. As shown in Fig. 6, infiltration by predominantly inflammatory cells and interstitial pneumonia with severe hyperemia were observed in the lungs of the control mimic-infected pigs at 72, 120 and 168 h post infection (Fig. 6D1–D3). However, in the miRNA mimic-infected group, no obvious hyperemia in the lungs was observed at 72 h post infection (Fig. 6E1), and light hyperemia at 120 and 168 h post infection was observed (Fig. 6E2,E3). No obvious histopathological changes were observed in non-infected group (Fig. 6F1–F3). In addition, PRRSV was detected in the sera of control mimic-infected pigs from 3 to 14 days post infection but was observed in miRNA mimic-infected pigs from 5 to 10 days post infection by RT-PCR (data not shown). The viral titers in the lungs of control mimic- and miRNA-infected pigs were calculated to be 10^−2.25^ TCID_50_/ml and 10^−0.625^ TCID_50_/ml at 168 h post infection, respectively (data not shown). Taken together, the results indicate that the ssc-miR-30d_R-1 mimic has a significant inhibitory effect on viral replication in PRRSV-inoculated SPF piglets.

## Discussion

miRNAs function mainly through repressing the expression of the targets^13^,^14^. Cellular miRNAs may regulate viral replication by targeting a sequence in the viral genome^15^,^16^,^17^,^18^,^19^,^20^,^21^,^22^,^23^ and/or regulate the cellular pathways involved in the viral life cycle^24^. The abundantly expressed miRNAs appeared to be more important than those expressed at relatively low levels. In this study, the results indicated that only a few miRNAs were abundantly and differently expressed in the lung tissues of infected and control pigs; however, they account for a large part of the miRNomes.

The data from this study demonstrate that ssc-miR-30d_R-1 is a novel antiviral host factor against PRRSV. ssc-miR-30d_R-1 reduced production of PRRSV progeny. In contrast, inhibition of ssc-miR-30d_R-1 substantially enhanced PRRSV propagation in both MARC-145 cells and PAMs, the main target cell types for PRRSV replication *in vivo*, confirming the biological relevance of this finding. Because targeting host factors for developing antiviral drugs has the advantage of a higher genetic barrier to the emergence of viral escape mutants, the identification and characterization of ssc-miR-30d_R-1 as an inhibitor of PRRSV replication may suggests new options for controlling future PRRS outbreaks, as current control measures remain inadequate.

In the present study, the reduction of PRRSV replication by ssc-miR-30d_R-1 did not appear to involve direct targeting of the PRRSV genome. However, these data indicated that ssc-miR-30d_R-1 might act on a proviral cellular factor(s)/pathway(s) to reduce PRRSV replication. Although NF-κB has long been considered a key transcription factor for the expression of a variety of antiviral cytokines^25^, some pathogens redirect the activity of NF-κB into a virus-supportive function^26^,^27^. For example, influenza viruses replicated to higher titers in cells with pre-activated NF-κB, and conversely, progeny virus production was reduced when NF-κB signaling was impaired^28^,^29^. Williams *et al.* reported that sustained induction of NF-κB is required for efficient expression of latent HIV type 1^30^. We show in this study that optimal replication of PRRSV, a virus from the *Arteriviridae* family, also relies on NF-κB.

Data on the interactions between PRRSV and the NF-κB pathway have also been somewhat controversial. Lee *et al.*^12^ first demonstrated that PRRSV infection activated NF-κB signaling in MARC-145 cells and PAMs via IkB degradation and p65 nuclear translocation. Previous studies also showed that PRRSV infection triggered NF-κB activation^31^, and the nucleocapsid (N) protein of PRRSV could activate NF-κB when ectopically expressed in MARC-145 cells^32^. However, the activated NF-κB could only be detected after 24 h post infection. In contrast, the ectopic expression of several individual PRRSV nsp proteins, such as nsp1a, nsp1b, nsp2, and nsp11, were reported to negatively regulate NF-κB activation. For example, Sun *et al.* reported that PRRSV nsp2 inhibited the NF-κB signaling pathway by interfering with the polyubiquitination process of IkBa^33^. Furthermore, PRRSV nsp1a could inhibit NF-κB activation and suppress IFN-β production^12^,^31^,^34^.

Taken together, it is reasonable to conclude that PRRSV activates NF-κB at late phases of infection and that PRRSV may have developed sophisticated strategies to either activate or inhibit NF-κB for its own benefit at different stages of its life cycle. The elaborate mechanisms by which PRRSV regulates NF-κB activation and how the latter promotes PRRSV replication require further study.

We showed that ssc-miR-30d_R-1 was decreased following infection in MARC-145 and PAM cells in the present study. We compared the basal expression levels of ssc-miR-30d_R-1, ssc-miR-27b, ssc-miR-145_R-1, ssc-miR-23a_R + 1, and ssc-miR-199a-5p in PAMs by qPCR and found that ssc-miR-30d_R-1 was among the most highly expressed miRNAs examined (data not shown). Interestingly, we found that PRRSV infection downregulated the expression of ssc-miR-30d_R-1 as the infection progressed. Significant downregulation was first observed at 12 h, and further reductions in ssc-miR-30d_R-1 levels were observed at later time points. It is plausible to speculate that PRRSV infection gradually decreases ssc-miR-30d_R-1 mRNA expression, which in turn, relieves the stabilizing effect on TLR4 mRNA, ultimately leading to subsequent NF-κB activation. In our study, ssc-miR-30d_R-1 can significantly inhibit the levels of pro-inflammatory cytokines (IL-8, IL-6, TNF-α and IL-1β) in a time-dependent manner in MARC-145 cells. Furthermore, we explored the effects of ssc-miR-30d_R-1 on PRRSV infection in SPF piglets by evaluating body temperature, body weight, viral titer, and pathological changes, among others. Although there was no significant difference in lung pathological changes between the miRNA mimic-infected group and control mimic-infected group after 168 h post infection, these results showed that treatment with ssc-miR-30d_R-1 mimics can significantly reduce the damage to infected piglets at an early stage. In addition, a previous study showed that the Toll-like receptor (TLR) signaling cascade may be involved in PRRSV-induced NF-κB activation^35^.

In summary, our data demonstrate that ssc-miR-30d_R-1 is an antiviral host factor that restricts PRRSV replication. Instead of directly targeting the PRRSV genome, ssc-miR-30d_R-1 exerts it antiviral effect by negatively regulating cellular NF-κB signaling, which we have shown to be a proviral factor for PRRSV replication. As a survival strategy, PRRSV downregulates the expression of ssc-miR-30d_R-1 post infection and activates NF-κB to facilitate its replication.

## Material and Methods

### Viral strain propagation

The highly pathogenic PRRSV (LS-4 strain) was used throughout the present study^36^,^37^. The viral strain was passaged five times in MARC-145 cells. The MARC-145 cell line was purchased from the American Type Culture Collection (ATCC no. CRL-1223) and cultured in DMEM (Invitrogen) supplemented with 10% fetal bovine serum (FBS), 100 U/mL penicillin and 100 mg/mL streptomycin in a humidified 37 °C/5% CO_2_ incubator.

Porcine alveolar macrophages (PAMs) were obtained by lung lavage from specific pathogen-free piglets as previously described^38^. All PAM batches were tested for susceptibility to PRRSV infection, and only highly PRRSV-susceptible cells were used for the present study. Prior to use, PAMs were suspended at 3 × 10^6^ cells/ml in EMEM with 5% FCS. For infection assays, PAMs in a non-adherent state were used. A total of 200 μL of cell suspension was added to sterile 1.5 mL Eppendorf polypropylene tubes, the tube caps were pierced with a 16 G needle to allow air exchange and cultures were incubated at 37 °C in a humidified 95% air + 5% CO_2_ atmosphere.

### Pig infections

Lung tissue samples were obtained from mock-infected and PRRS virus-infected pigs at 24 h post infection and immediately snap-frozen in liquid nitrogen and stored at −80 °C until use. In brief, specific-pathogen-free (SPF) 4-week-old, Large White × Duroc crossbred weaned piglets (n = 12) were obtained from a swine herd at the Beijing Center for SPF Swine Breeding & Management. Piglets were randomly assigned to two groups: the PRRS virus infection group (n = 9) and the mock-infection control group (n = 3). Piglets in the PRRSV-infected group were aseptically inoculated intranasally with 2 mL (3 × 10^4^ TCID_50)_ and intramuscularly with 1 mL (2 × 10^4^ TCID_50_)^36^. After PRRSV inoculation, piglets were monitored for the clinical signs of infection as described previously^39^. Food and water were available ad libitum. Piglets were humanely sacrificed as necessary to ameliorate suffering. All experiments were performed in accordance with the National Institute of Health Guide for the Care and Use of Laboratory Animals (NIH Publications No. 80–23) and were approved by the Animal Care and Use Committees of the Institute of Zoology, Chinese Academy of Sciences. Efforts were made to minimize suffering and to reduce the number of animals used.

### Total RNA isolation and Illumina Solexa MPSS

Total RNA (virus-infected group, n = 3; mock-infected group, n = 3) was extracted using the miRNeasy Mini Kit (Qiagen 217004, Germany) according to the manufacturer’s protocol. RNA quality and quantity for Solexa sequencing were evaluated using the Agilent 2100 Bioanalyzer. Sequencing was performed according to Chen *et al.*^40^. Sequencing data were analyzed using SOAP (short oligonucleotide alignment program) following Li *et al.*^41^. Clean sequenced reads (excluding reads containing ambiguous base and adaptor contaminants) yielded by Solexa sequencing were used for further analysis. After removal of reads classified as small RNAs from rRNA and other species (rRNA, tRNA, scRNA, snRNA, and snoRNA), the length distribution of miRNA-mappable data by reads and family was annotated and calculated (data not shown).

Sequenced sequences were processed using Illumina’s Genome Analyzer Pipeline software and then subjected to a series of data filtration steps, using the mammalian miRNA data in miRBbase v16.0, to obtain mappable sequences with the ACGT101-miR program. The merged reference database of the pig genome (2.26 Bbp) (Sscrofa9 ftp://ftp.sanger.ac.uk/pub/S_scrofa/assemblies/) and non-redundant ESTs (0.5 billion nt) (ftp://ftp.ncbi.nih.gov/repository/dbEST) were constructed as an available complete sequence database for pig (named the pig genome and EST) and used for mapping.

### Reagents and antibodies

Antibodies specific to Toll-like receptor 4, MyD88 and NF-κB p65 were from Cell Signaling Technology (Danvers, MA). Antibodies specific to β-actin and horseradish peroxidase-coupled secondary antibodies were obtained as described previously^42^,^43^,^44^. Cholesterol-conjugated ssc-miR-30d-R_1 mimic and TLR4 siRNA for *in vivo* RNA delivery, ssc-miR-30d-R_1 mimic and inhibitor for *in vitro* transfection and their respective negative controls were from Ribobio Co. (Guangzhou, China)^45^.

### qPCR analysis

miRNA expression in PAMs from mock- or viral-infected lung tissues was assayed at 24, 48 and 72 h post infection. Total RNA was extracted from PAMs with TRIzol reagent and purified according to the manufacturer’s recommendation (Invitrogen) and used for miRNA expression assays. Correspondingly, miRNA expression was evaluated in mock- or virus-infected MARC-145 cells at the same time points. In addition, mRNA levels of the pro-inflammatory cytokines IL-1β, IL-6, IL-8 and TNF-α in MARC-145 and PAM cells from lung tissues were measured at different time points. Cellular β-actin or GADPH mRNA from the same RNA extract was used as internal control. A total of 2 μg of the RNA was converted to cDNA using random primers in a 20 μL reaction and then amplified using SYBR Green Master Mix following the manufacturer’s protocol (Vazyme, China). The primer sequences for miRNA and targeted cytokines as well as β-actin and GADPH are listed in the supporting materials (Tables S1 and S2).

### ssc-miR-30d_R-1 target prediction analysis

Potential target genes for selected significant differentially expressed miRNAs were identified using the *Sus scrofa* Unigene database (NCBI) and the miRanda algorithm (version 3.3; http://www.microrna.org) with the following parameter settings: score threshold >130 and free energy threshold <−16 kCal/mol. The list of potential target genes was further filtered using the following higher stringency methods: (1) a match between nucleotides 2–8 of the miRNA with the target sequence or (2) a match between nucleotides 2–7 and 13–16 of the miRNA with the target sequence (G:U wobble tolerance) and (3) miRNA binding sites must lie within the 3′-UTR. For each potential target gene, the 3′-UTR flanking the miRNA binding site(s) was PCR-amplified from pig genomic DNA using gene-specific primers (Table S3). Each PCR product from 13 target genes was subcloned into the pMIR-REPORT vector downstream of the luciferase ORF to generate the reporter vectors pMIR-CD4, pMIR-NFYB, pMIR-RANBP9, pMIR-NFAT5, pMIR-TLR4, pMIR-CD53, pMIR-IL1A, pMIR-CD8A, pMIR-EPCAM, pMIR-IRG6, pMIR-IL12A, pMIR-IL12B, and pMIR-MMD. All cDNA constructs were verified by DNA sequencing.

To determine whether ssc-miR-30d_R-1 can target the viral segments, the pMIR-REPORT luciferase reporter vector (Ambion) was used as the cloning vector for a reporter gene assay analyzing the potential target region of ssc-miR-30d_R-1 in the PRRSV genome according to the method reported previously^8^. The 21 cDNA fragments corresponding to the 5′-UTR, 3′-UTR, and 19 nonstructural and structural genes (nsp1a, nsp1b, nsp2-nsp5, nsp7-nsp12, ORF2a, ORF2b, ORF3-ORF7) of PRRSV were amplified by PCR from PRRSV RNA (LS-4 strain) and subcloned into the pMIR-REPORT vector downstream of the luciferase ORF to generate the reporter vectors pMIR-5′-UTR, pMIR-3′-UTR, pMIR-nsp1a, pMIR-nsp1b, pMIR-nsp2, pMIR-nsp3, pMIR-nsp4, pMIR-nsp5, pMIR-nsp7, pMIR-nsp8, pMIR-nsp9, pMIR-nsp10, pMIR-nsp11, pMIR-nsp12, pMIR-ORF2a, pMIR-ORF2b, pMIR-ORF3, pMIR-ORF4, pMIR-ORF5, pMIR-ORF6, and pMIR-ORF7, respectively. The primers used are listed in Table S3. All cDNA constructs were verified by DNA sequencing.

### Transfection and dual-luciferase activity assay

MARC-145 cells were seeded into 6-well plates and incubated overnight. The indicated plasmids and miRNA mimics or inhibitors were transfected into cells using Lipofectamine 2000 (Invitrogen, Carlsbad, CA) according to the manufacturer’s protocol. Briefly, 24 h after transfection, firefly and *Renilla* luciferase activities were measured using a dual-luciferase assay kit (Promega) with a plate reader (PerkinElmer, MA, USA). The *Renilla* and firefly luciferase signals were detected using the Veritas Microplate Luminometer (Turner Biosystems, Sunnyvale, CA, USA). The firefly luciferase signal was normalized to the *Renilla* luciferase signal. The normalized firefly luciferase activity was compared between ssc-miR-30d_R-1 mimic and the control mimic cells. The results are expressed as relative activity. Each target construct was tested in triplicate, and the assay was repeated to confirm the results.

### Western blotting

MARC-145 cells were transfected with the ssc-miR-30d_R-1 mimic or the control mimic and inhibitor TLR4 siRNA prior to PRRSV infection and LPS. Cells were collected at 24^th^ day post infection and lysed by adding 250 μL 2 × lysis buffer A (LBA; 65 mM Tris-HCl, pH 6.8, 4% sodium dodecyl sulfate, 3% DL-dithiothreitol and 40% glycerol). Cell lysates were then analyzed for expression of TLR4, MyD88 and NF-κB by Western blotting using a specific monoclonal antibody (MAb) as the primary antibody (1:1000). β-actin was detected with an anti-β-actin monoclonal antibody (MAb) (Beyotime, China) to normalize the protein content of the samples.

### *In vitro* study

The ssc-miR-30d_R-1 mimic or a control mimic was transfected into MARC-145 cells using Lipofectamine 2000 (Invitrogen) according to the manufacturer’s instructions. Treated MARC-145 cells were infected with PRRSV virus at a multiplicity of infection (MOI) of 0.1 at 37 °C. After one hour of incubation, the cells were washed with warm PBS and incubated in DMEM medium supplemented with 100 U/ml penicillin, 100 μg/ml streptomycin, 2 μg/mL TPCK-treated trypsin and 0.2% bovine serum albumin (fraction V). Supernatants were collected after 24 h and stored at −70 °C for the TCID_50_ assay. To determine whether the virus replicated in a dose-dependent manner in cells, MARC-145 and PAM cells were transfected with ssc-miR-30d_R-1 mimic or a control mimic at the indicated dose (50, 100, 150 and 200 ng), followed by PRRSV infection (MOI = 0.1).

### *In vivo* assay

To study the antiviral effects of ssc-miR-30d_R-1 in piglets, each group of 12 piglets was intravenously injected with a 200 μL volume of PBS (as a control group, non-infection), synthetic ssc-miR-30d_R-1 (0.1 nmol) or control mimic (0.1 nmol) per day for 8 days. On day 2, these piglets were inoculated intranasally with a highly pathogenic PRRSV strain (LS-4 strain) at a 2 mL 10^5^ TCID_50_ dose for each piglet in the mimic-treated group. Eighteen pigs (n = 3 in each time point) were slaughtered at 72, 120 or 168 h post infection. Two portions from each lobe of the left lung were collected immediately and fixed in 4% paraformaldehyde solution. The whole left lung was weighed before and after desiccation at 80 °C, drying to a constant weight, and then the lung wet:dry weight ratio was determined, which was used as one indicator of lung edema^46^. Lung histopathological changes at 72, 120 and 168 h post infection were analyzed based on previously described reports^47^. According to the method of Reed-Muench, virus titration in the lungs was determined, with some modification. Briefly, lung tissues were sampled, frozen in liquid nitrogen and then ground into fine powder using a mortar and pestle, weighted for one milligram and homogenized in 1 mL cold phosphate-buffered saline. Clarified homogenates were titrated for viral infectivity in MARC-145 cells cultured in 96-well plates from initial dilutions of 1:10. Viral titers were expressed as the mean TCID_50_/mL. The remaining piglets (n = 3) were monitored for 14 days for virus detection in serum.

### Statistical analysis

Data extracted from 3 independent experiments were expressed as the mean ± standard deviation. Statistical relationships were assessed using Student’s t test, considering *P* values < 0.01 (**) and < 0.001 (***) as significant.

## Additional Information

**How to cite this article**: Wang, C. *et al.* Identification of miRNomes reveals ssc-miR-30d-R_1 as a potential therapeutic target for PRRS viral infection. *Sci. Rep.* **6**, 24854; doi: 10.1038/srep24854 (2016).

## Supplementary Material

Supplementary Information

## Figures and Tables

**Table 1:** Most abundantly expressed miRNAs in PRRSV infection and matched normal lung tissues.

miR_name	miR_seq	TPM in normal lung			TPM in infected lung			Normal/infection
		Sample 1	Sample 2	Sample 3	Sample 1	Sample 2	Sample 3	
ssc-miR-30d_R-1	TGTAAACATCCCCGACTGGAAGC	3345	3454	3521	1039	1004	996	3.40
ssc-miR-27b	TTCACAGTGGCTAAGTTCTGC	21271	23567	22453	9807	10002	10244	2.24
ssc-miR-145_R-1	GTCCAGTTTTCCCAGGAATCCCT	5619	5746	5531	2836	3021	2968	1.91
ssc-miR-23a_R + 1	ATCACATTGCCAGGGATTTCCA	2447	2563	2451	1357	1532	1520	1.69
ssc-miR-199a-5p	CCCAGTGTTCAGACTACCTGTTC	3755	3852	3732	2322	2531	2438	1.56
ssc-miR-99a_R-1	AACCCGTAGATCCGATCTTGT	1625	1707	1797	1161	1066	1089	1.55
ssc-miR-199b_R + 1	CCCAGTGTTTAGACTATCTGTTC	1022	918	993	487	401	454	2.19
ssc-miR-133a-3p_R + 1	TTGGTCCCCTTCAACCAGCTGT	832	632	682	387	309	325	2.1
ssc-miR-423-3p	AGCTCGGTCTGAGGCCCCTCAGT	643	549	742	328	303	287	2.1
ssc-miR-374b-5p	ATATAATACAACCTGCTAAGTG	299	277	214	165	119	138	1.87
ssc-miR-532-5p	CATGCCTTGAGTGTAGGACCGT	558	494	662	311	308	285	1.90
hsa-miR-181a*	ACCATCGACCGTTGATTGTACC	228	185	208	105	84	69	2.41
hsa-miR-148a*	AAAGTTCTGAGACACTCCGACT	362	282	212	209	190	198	1.43
hsa-miR-221*_R + 4	ACCTGGCATACAATGTAGATTTCTGT	99	112	94	213	181	157	1.36
ssc-miR-221	AGCTACATTGTCTGCTGGGTTT	105	125	111	257	204	217	1.99
hsa-miR-34c-3p_L-1	ATCACTAACCACACGGCCAGG	86	61	72	187	161	193	2.47
PN-ssc-miR-1307-5p	TCGACCGGACCTCGACCGGCT	224	187	155	407	383	353	2.02
PN-ssc-miR-24_R-3	TGGCTCAGTTCAGCAGGAA	181	142	111	396	353	341	2.51
hsa-miR-449a	TGGCAGTGTATTGTTAGCTGGT	292	281	201	658	622	617	2.45

miRNAs with TPM >100 and more than a 1.5-fold alteration are listed, but miRNAs with TPM > 1000 and more than a 1.5-fold alteration were considered most likely to be important in PRRSV pathogenesis in this study.
