# Supplementary material for: Identification of miRNomes reveals ssc-miR-30d-R_1 as a potential therapeutic target for PRRS viral infection
Source: Sci Rep. 2016 Apr 27;6:24854. doi: 10.1038/srep24854 (PMC4846818; doi:10.1038/srep24854)
Supplement: Supplementary Information [file srep24854-s1.pdf]

Supplementary Information for "Identification of miRNomes reveals ssc-miR-30d-R_1 as a potential therapeutic target for PRRS viral infection"

Chengmin Wang[1$],Yanyu Zhang[2$],Jing Luo[1$],Hua Ding[3],Shelan Liu[4], Said Amer[5], Li xie[3],Wenting Lyv[1],Wen Su[1],Meng Li[1],Qinmiao Sun[1],Jiayin Dai[1] and Hongxuan He[1]*

[1]Key Lab of Animal Ecology and Conservation Biology, Institute of Zoology, Chinese Academy of Sciences, Beijing, 100101, P.R China;

[2]Beijing Institute of Transfusion Medicine, Academy of Military Medicine Sciences, Beijing, 100850, P.R China;

[3]Department of Infectious Disease, Hangzhou Center for Disease Control and Prevention, Hangzhou, Zhejiang Province,310021,P.R China;

[4]Department of Infectious diseases, Zhejiang Center for Disease Control and Prevention, Hangzhou, Zhejiang Province, 310051, P.R China;

[5]Department of Zoology, Faculty of Science, Kafr El sheikh University, Kafr El sheikh 33516, Egypt

[$]These authors contributed equally to this paper.

*Authors for correspondence: hehx@ioz.ac.cn

Fig. S1. ssc-miR-30d_R-1 does not directly target the PRRSV genome.

MARC-145 cells transfected with construct encoding wild-type (WT) or mutated (Mut) viral gene 3`-UTR plus mimics. MARC-145 cells were co-transfected with 0.1 mg of indicated constructing luciferase reporter, 0.05 mg of pRL-TK, and 30 nM of ssc-miR-30d_R-1 mimic or negative control of mimic. At 24 h post-transfection, cells were lysed for dual-luciferase assay.The relative luciferase activities (ssc-miR-30d_R-1/NC) refer to fold change in luciferase activity in ssc-miR-30d_R-1 mimic-transfected cells relative to respective NC mimic-transfected controls. *P < 0.05 and **P < 0.01 (Student's t-test). Data are from three independent experiments (mean±SD).

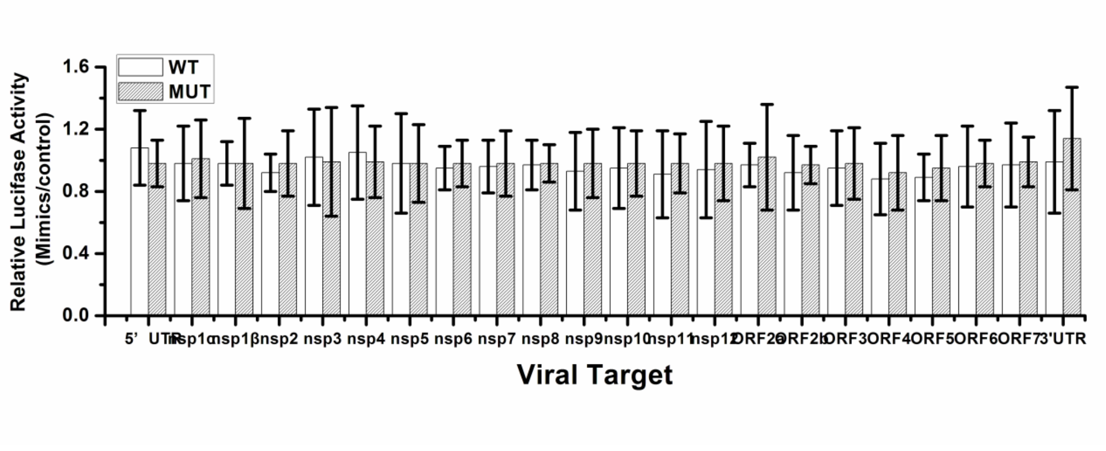

Fig. S2. The suppressive effect of ssc-miR-30d_R-1 on pro-inflammatory cytokines. A-D indicates ssc-miR-30d_R-1 mimic and control mimic in PAM cells. ssc-miR-30d_R-1 mimic reduced mRNA expression level of pro-inflammatory (IL-8, IL-6, TNF-α and IL-1β) in a time-dependent manner in MARC-145 cells. E-H indicates ssc-miR-30d_R-1 inhibitor enhanced mRNA expression level of

pro-inflammatory (IL-8, IL-6, TNF-α and IL-1β) in a time-dependent manner in MARC-145 cells (F). MARC-145 were transfected with mimic or inhibitor and followed by PRRSV infection (MOI = 0.1). Data are expressed as means standard deviations of three independent experiments. P values were calculated using Student's t test. *P<0.05; **P<0.01;*** P<0.001. Similar results in PAM cells were shown in Fig.3.

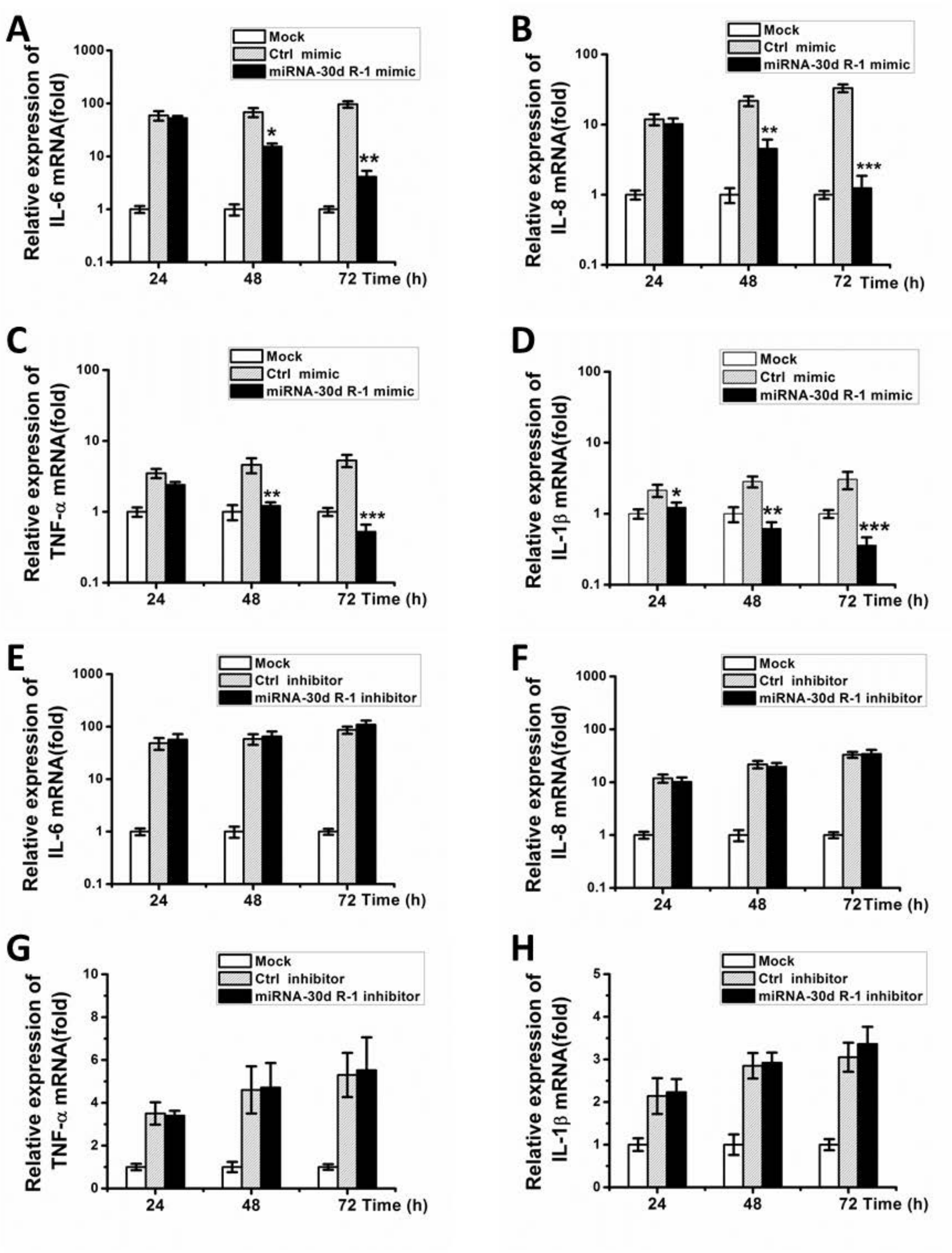

Table S1.The primers of cytokines mRNA used for Q-PCR.

	Name	Forward Primer	Reversed Primer	Probe
**MARC-145**	β-actin	TGACTGACTACCTCATGAAGATCC	TCTCCTTAATGTCACGCACGATT	CGGCTACAGCTTCACCACCACGGC
	IL-6	AGAGGCACTGGCAGAAAAC	TGCAGGAACTGGATCAGGAC	
	IL-8	AGGACAAGAGCCAGGAAGAA	ACTGCACCTTCACACAGAGC	
	IL-1β	GGAAGACAAATTGCATGG	CCCAACTGGTACATCAGCAC	
	TNF-α	TCTGTCTGCTGCACTTTGGAGTGA	TTGAGGGTTTGCTACAACATGGGC	
	GADPH	CGGAGTCAACGGATTTGGTCGTA	AGCCTTTCTCCATGGTGGTGAAGAC	
**PAM**	IL-6	CCTTCAGTCCAGTCGCCTTCTCC	GCATCACCTTTGGCATCTTCTTCC	
	IL-8	CACTGTGAAAATTCAGAAATCATTGTTA	CTTCACAAATACCTGCACAACCTTC	
	IL-1β	ACCTGGACCTTGGTTCTCTG	CATCTGCCTGATGCTCTTGT	
	TNF-α	TGGTGGTGCCGACAGATGG	GGCTGATGGTGTGAGTGAGGAA	
	GADPH	TGCCAACGTGTCGGTTGT	TGTCATCATATTTGGCAGGTTT	

Table S2. Primers used were shown in abundantly expressed miRNAs in PRRSV infection and normal cells.

**miRNA name**	**miRNA Sequence(5' To 3')**	**RT primer(5' To 3')**	**Forward Primer(5' To 3')**	**Reversed Primer(5' To 3')**	**PCR product Length (bp)**
Ssc-miR-27b	UUCACAGUGGCUAAGUUCUGC	GTCGTATCCAGTGCAGGGTCCGAGGTATTCGCACTGGATACGACGCAGAA	GGCCGTTCACAGTGGCTAAG	CAGTGCAGGGTCCGAGGTAT	62
Ssc-miR-30d_R-1	UGUAAACAUCCCCGACUGGAAGC	GTCGTATCCAGTGCAGGGTCCGAGGTATTCGCACTGGATACGACG	TGCCGGCTGTAAACATCCC	CAGTGCAGGGTCCGAGGTAT	66

		CTTCC			
Ssc-miR-145_R-1	GUCCAGUUUUCCCAGGAAUCCCU	GTCGTATCCAGTGCAGGGTCCGAGGTATTCGCACTGGATACGACAGGGAT	TTGCTGCTGTCCAGTTTTCC	CAGTGCAGGGTCCGAGGTAT	67
Ssc-miR-23A_R+1	AUCACAUUGCCAGGGAUUUCCA	GTCGTATCCAGTGCAGGGTCCGAGGTATTCGCACTGGATACGACTGGAAA	TGGCTGATCACATTGCCAG	CAGTGCAGGGTCCGAGGTAT	64
Ssc-miR-199A-5p	CCCAGUGUUCAGACUACCUGUUC	GTCGTATCCAGTGCAGGGTCCGAGGTATTCGCACTGGATACGACGAACAG	GCTGGCCCAGTGTTCAGACTA	CAGTGCAGGGTCCGAGGTAT	64
TLR 4			TCAGTTCTCACCTTCCTCCTG	GTTCATTCCTCACCCAGTCTTC	166

Table S3. The predicted targets of ssc-miR-30d_R-1.

Predicted Target	Primer Sequence	
	Forward	Reverse
CD4	GTCAGAGCTCCTTGCTTCTGCTTTTCCAG	GTCATCTAGACCTGTGCTTCATGCTTCAGA
NFYB	GTCAGAGCTAAATGGTGCCCCTTTCATTT	GTCATCTAGAACGTTTCTTGGTTCCCACAG
RANBP9	GTCAGAGCTCGTTCAAATTGATCATCACAAA	GTCATCTAGAGAGCGGGGGTCAGAATATC
NFAT5	GTCAGAGCTCCACACTTACAAACTGGGAACA	GTCATCTAGACCATACATTACCCCCTTCATT
TLR4	GTCAGAGCTCCTGGTGATTCAGTGATGCCC	GTCATCTAGAATCAGGAGCCTTCTGATTTA
CD53	GTCAGAGCTAAATCATGCCCAGTCCACTC	GTCATCTAGATGAAACCTCACCTTTGGAGAA
IL1A	GTCAGAGCTTGTCAACTCCTTGTGATCTCTG	GTCATCTAGACAGGGCTCCCAGTTACTAGAAT
CD8A	GTCAGAGCTTCACACACACAACGGTGAAG	GTCATCTAGACCTCAGCCTCATTCCTTTGT
EPCAM	GTCAGAGCTCCAGCATTTGGACTGCATAG	GTCATCTAGATCAAAGTAAACAGAAAGGCAAACA
IRG6	GTCAGAGCTCCCCTGTATTTCCATTTTGAAG	GTCATCTAGAATCTGAGGGCAGACAAGGAA
IL12A	GTCAGAGCTAAAAATTGGAACCAAA	GTCATCTAGAGGGAGCCTCACATCT

	GAAATG	TTCAA
IL12B	GTCAGAGCTTTTGAGGAGGTCCTGGAGAG	GTCATCTAGAGGTGGGGAGTGAAGTTGAAT
MMD	GTCAGAGCTTCCCCCTCACAGCAAATAAG	GTCATCTAGAGCACTGACTTTCAACCTGACC
5’UTR	GCGACTAGTATGACGTATAGGTGTTGGC	ATAAAGCTTTGGTTAAAGGGGTGGAGAG
nsp1α	GCGGAGCTCATGTCTGGGATACTTGATCGGTGCAC	ATAAAGCTTCTGCGGGAGCGGCAAGTTGGTTAAC
nsp1β	GCGACTAGTAGGCCCAAACCTGAGGACTTTTGCCC	ATAAAGCTTACCGTACCACTTATGACTGCCAAACC
nsp2	ACAACTAGTGCCGGAAAGAGAGCAAGGAAAACACG	ACAACGCGTGCCCAGTAACCTGCCAAGAATGGCAA
nsp3	GCGACTAGTGGGGCACGCTACATCTGGCACTTT	ATAAAGCTTCTCAAGGAGGGACCCGAGCTGAGA
nsp4	ATAACTAGTGGCGCTTTCAGAACTCAAAAGCCCTC	ATAAAGCTTTTCCAGTTCGGGTTTGGCAGCAAGCA
nsp5	ATAACTAGTGGAGGCCTTTCCACAGTTCAACTTCT	GCGAAGCTTCTCGGCAAAGTATCGCAAGAAGAAAG
nsp6	GCGACTAGTATGACGTATAGGTGTTGGC	ATAAAGCTTTGGTTAAAGGGGTGGAGAG
nsp7	ATAACTAGTTCGCTGACTGGTGCCCTCGCCATGAG	GCGAAGCTTTTCCCACTGAGCTCTTCTATTCTCG
nsp8	ATAACTAGTGCCGCCAAGCTTTCCGTGGAGCAAG	GCGACGCGTCTAGCAGTTTAAACACTGCTCCTTAG
nsp9	ACAACTAGTGGAGCAGTGTTTAAACTGCTAGCCGC	GAGAAGCTTCTCATGATTGGACCTGAGTTTTTCCC
nsp10	GCGACTAGTGGGAAGAAGTCCAGAATGTGCGGGTA	ATAAAGCTTTTCCAGGTCTGCGCAAATAGCGCGGA
nsp11	ATAACTAGTGGGTCGAGCTCCCCGCTCCCCAAG	GCGAAGCTTTTCAAGTTGGAAATAGGCCGTCTTG
nsp12	AGAACTAGTGGCCGCCATTTTACCTGGTATCAACT	TCAAAGCTTTCAATTCAGGCCTAAAGTTGGTTCAA
ORF2a	GAGACTAGTATGAAATGGGGTCTATGCAAAGCCTC	GAGACGCGTTCACCATGAGTTCAAAAGAAAAGTTG
ORF2b	TATACTAGTATGGGGTCTATGCAAAGCCTCTTTGA	GCGAAGCTTTCATAAGATCTTCTGTAATTGCTCAG
ORF3	GGCACTAGTATGGCTAATAGCTGTACATTCCTCCA	TCAAAGCTTCTATCGCCGTGCGGCACTGAGAAATT
ORF4	GGCACTAGTATGGCTGCGTCCTTTCTTTTCCTCTT	TCGAAGCTTTCAAATTGCCAGTAGGGATGGCAAAA
ORF5	ATAACTAGTATGTTGGGGAAGTGCTTGACCGCGTG	TAAAAGCTTCTAGAGACGACCCCATAGTTCCGCTG
ORF6	ATAACTAGTGAACTATGGGGTCGTC	GGCAAGCTTCTTGCCGTTGTTATTTG

	TCTAG	GCAT
ORF7	GGCACTAGTATGCCAAATAACAACGGCAAGCAG	TAAAAGCTTTCATGCTGAGGGTGATGCTGTGGC
3'UTR	GGCGAGCTCTGGGCTGGCATTCTTT	GGCAAGCTTTTAATTACGGCCGCATGG

UTR, untranslated region
